# Exploring fluorescent light energy as management option for canine superficial bacterial folliculitis

**DOI:** 10.3389/fvets.2023.1155105

**Published:** 2023-06-02

**Authors:** Andrea Marchegiani, Andrea Spaterna, Alessandro Fruganti, Matteo Cerquetella

**Affiliations:** School of Biosciences and Veterinary Medicine, University of Camerino, Matelica, Italy

**Keywords:** superficial bacterial folliculitis, superficial pyoderma, dog, fluorescent light energy, topical therapy

## Abstract

Superficial bacterial folliculitis (SBF) represents a common dermatological diagnosis in dogs that can be successfully managed with either topical and/or systemic treatments. In the present study we evaluated the efficacy of a fluorescent light energy (FLE) device as sole management for SBF. The same FLE device has been shown, as adjunct therapy to systemic antibiotic or alone, to effectively control clinical manifestation of interdigital furunculosis. Twenty dogs were randomized to receive FLE once (six dogs) or twice (six dogs) weekly in comparison with oral anti-biotic (eight dogs) until complete healing. FLE regimen was able to significantly reduce the time needed to clinical resolution for oral antibiotic, supporting owners’ compliance and welfare of dogs.

## 1. Introduction

Canine pyoderma includes a number of diseases that involves bacterial skin infection characterized by different clinical and histopathological aspects. Among canine pyodermas, superficial bacterial folliculitis (SBF, or superficial pyoderma) consists in a bacterial infection that remained confined within hair follicles, without invasion of the dermis (1). *Staphylococcus pseudintermedius* is a normal inhabitant of canine skin and represents the principal contributing bacterium in canine pyodermas, including SBF (2). Clinical manifestation of SBF usually develops secondary to a range of diseases that causes cutaneous damage, immunocompromise and inflammation, including allergic, endocrine, seborrheic, and follicular disorders (3, 4). SBF may be successfully managed with topical antimicrobial treatment as the only treatment for superficial clinical lesions even if systemic antibiotic is also a widely used approach and optional for treatment of superficial pyoderma (5). Currently, different topical approach to manage SBF have been tested (6–9) due to the arisen multidrug resistance and the progressive restriction of veterinary antimicrobial drug use (10–12). Among non-pharmacological procedures explored in canine dermatology, promising results have been obtained using different forms of photobiomodulation, in which photons (mainly produced by light-emitting diodes lamps) are administered at different wavelengths to influence biological activity and enhance cutaneous restoration from diseases (13, 14). Fluorescent light energy (FLE) is an innovative photobiomodulation regimen that uses photons at different wavelengths and at non-thermal irradiance to influence several biological activities and promote cutaneous repair (13). It has been successfully tested for the management of pododermatitis (15) and deep pyoderma (16) in dogs, in addition to have been applied in a variety of dermatological disorders (14). The aim of the present study is to investigate the effect of FLE in dogs affected by SBF, to ascertain if FLE management alone can significantly reduce the time needed to achieve clinical resolution of the disease, in comparison with systemic antibiotic.

## 2. Materials and methods

### 2.1. Inclusion and exclusion criteria

The study protocol was compliant with European legislation on the protection of animals used for scientific purposes and approved by the University of Camerino Body for Protection of Animals (Prot. N. 1/2017). The dogs enrolled in the study were client-owned pets that were presented to the Veterinary Teaching Hospital of University of Camerino for investigation of SBF, exhibiting a range of clinical lesions including papules, follicular pustules, epidermal collarettes and serous crusts. For a definitive diagnosis of SBF, dogs underwent cytological examination taking sample from follicular pustules or under serous crust, if present. Samples were also taken for culture and susceptibility testing from all dogs at the time of enrolment. For qualitative bacteriological assessment, we used the same protocol described elsewhere (17). Briefly, each swab underwent a pre-enrichment using Tryptic Soy Broth (Liofilchem, Italy) and incubated at 37°C for 6-h. Then, each sample was spread onto Columbia Agar plate containing 5% sheep blood, with and without Streptococcus supplement, Mannitol Salt agar, and Mac Conkey agar (Liofilchem, Italy). Plates were incubated at 37°C for 24–72 h in aerobic conditions. Gram positive bacteria were identified by Gram staining, catalase and coagulase tests, colony morphology and using commercial biochemical gallery (Remel RapID, ThermoFisher, Milan, Italy). Gram negative bacteria were identified by Gram staining, oxidase testing and commercial biochemical gallery (Remel Rapid ID, ThermoFisher, Milan, Italy). The same bacteriological specimens were used for routine susceptibility testing, performed by the minimum inhibitory concentration (MIC) method (18) and dogs screened to be multiresistant were excluded. Skin scraping and cytology were performed before enrolment on all dogs to check for ectoparasites and *Malassezia* spp. infection. The identification of *Demodex canis* or *Malassezia* spp. on skin scrapings or cytology represented an exclusion criterion. Previous treatments with systemic antibiotics, antihistamines, glucocorticoids, ciclosporin, topical anti-inflammatory or antimicrobial therapy in the prior 2 weeks also represented exclusion criteria. Pregnant or lactating female or dogs aged less than 12 months were not enrolled. No study dogs were on lokivetmab or oclacitinib during the study period. The primary endpoint of the study was to evaluate time (in weeks) to complete clinical resolution, intended as the total disappearance of the lesions(s) initially present.

### 2.2. Dogs’ randomization and study interventions

Enrolled dogs were randomly allocated into three groups. Group A (eight dogs) received systemic antibiotic therapy alone (cefadroxil 20 mg/kg PO BID or other suitable molecule upon culture and sensitivity testing) for at least 2 weeks after achieving clinical resolution; Group B (seven dogs) received only fluorescent light energy (Phovia™, Vetoquinol, France) application once a week until clinical resolution; Group C (seven dogs) received solely twice weekly fluorescent light energy application (every three to 4 days), until complete clinical resolution.

As previously described (15), fluorescent light system consisted of applying an approximate 2 mm layer of the gel on the SBF affected area and illuminating with the LED lamp (blue light-emitting diode device that delivers noncoherent blue light with peak wavelength between 440 and 460 nm and a power density between 55 and 129 mW/cm^2^), for 2 min, at approximately 5 cm distance. After illumination, the gel was gently removed using sterile gauzes dipped in sterile saline solution. Dogs were kept by their owner during fluorescent light energy application, and no sedation or excessive containment was needed. Since the goal of the present study was to assess an effect of fluorescent light energy as solely management of SBF, topical antimicrobial solutions were not permitted in either group. Feeding and housing conditions were maintained on a consistent basis during the length of the study.

Time of healing of different groups were analyzed using one-way Analysis of Variance (ANOVA, Graphpad Prism 8) for group comparisons of normally distributed variables, considering significant values of *p* < 0.05.

## 3. Results

**Figure 1:**
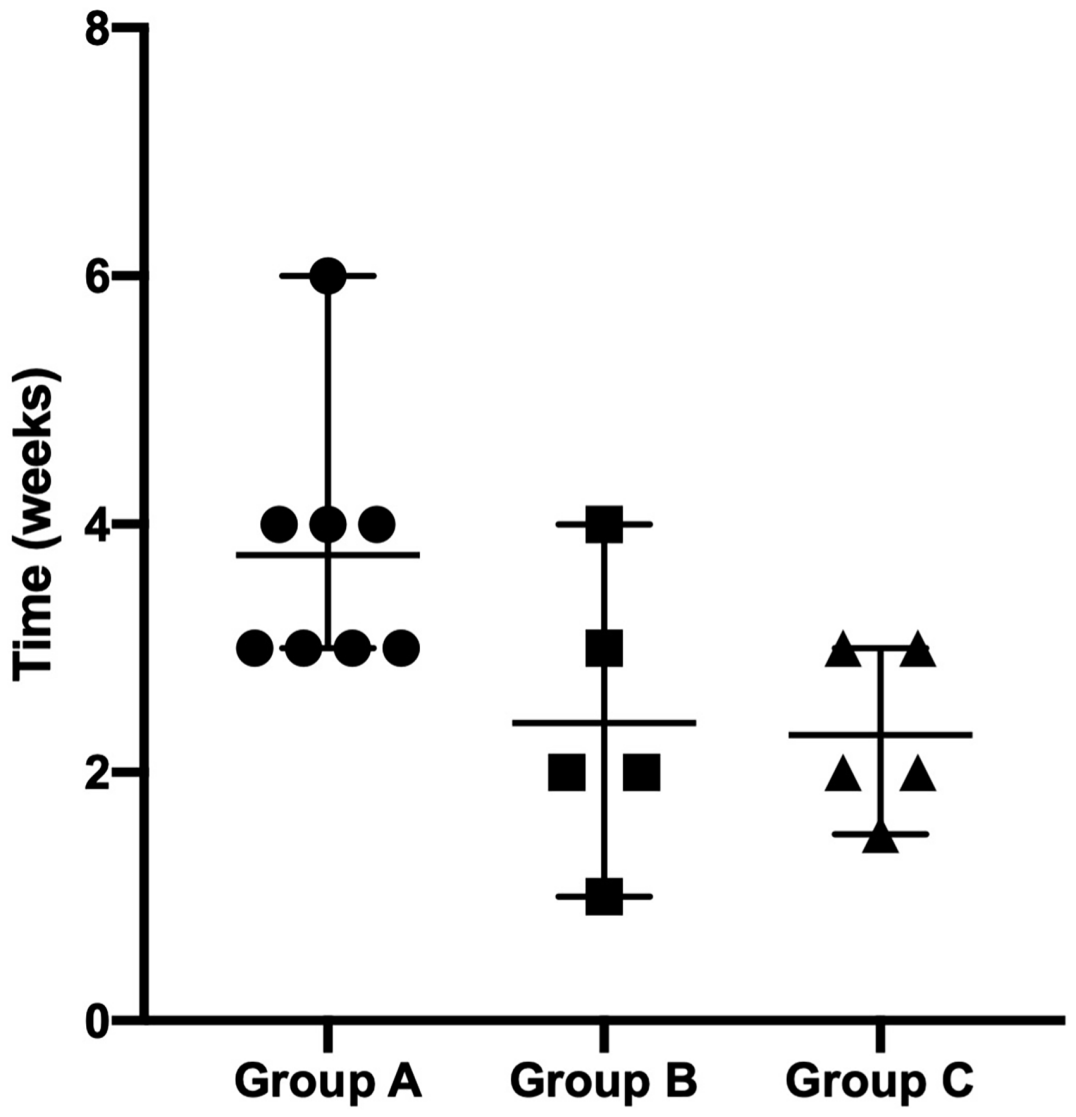
Average time to succeed complete clinical resolution in dogs suffering from SBF.

Two dogs (one in Group B and one in Group C) were prematurely withdrawn due to the owners’ inability to be compliant with the proposed fluorescent light energy scheme. Table 1 summarizes sex distribution, age, and body weight of enrolled dogs, which did not differ significantly. For all groups, there was no single case in which the dog’s condition worsened after treatment was initiated nor were there any adverse events detected by the clinician or reported by the dog’s owner. No SBF recurrence was reported in the first 6 months after complete clinical healing. Cytological evaluation confirmed bacterial follicular involvement in all cases. Microbiological swabs performed on lesions revealed the predominant presence of *Staphylococcus pseudintermedius* in all cases and other bacteria were found in coculture (Table 2). In group A, no antibiotic change was needed as cefadroxil was susceptible in all the bacterial isolates. Average time to achieve complete clinical resolution was equal to 3.75 ± 1.04, 2.40 ± 1.14, and 2.30 ± 0.67 weeks for group A, B, and C, respectively (Figure 1). Statistical analysis revealed a significant difference (*p* = 0.0291) in time needed to achieve complete clinical resolution from SBF.

**Table 1:** Signalment data of dogs that concluded the study.

	Group A	Group B	Group C
Breed (n)			
Bolognese		1	
English bulldog		2	
English setter	2		
French bulldog			1
German shepherd	1	1	1
Irish setter	1		
Italian bloodhound	1		
Pittbull			1
Shi-tzu		1	
Mixed breed	3	1	3
Age (years)			
Mean	5.06	5.67	4.92
sd	1.40	1.37	2.76
Sex (n)			
Male	4	3	3
Female	4	3	3
Weight (kg)			
Mean	21.13	21.17	21.00
sd	6.92	13.04	12.28
BCS			
mean	5.38	5.33	5.50
sd	0.74	0.52	0.55

**Table 2:** Bacterial strains isolated from superficial bacterial folliculitis lesions.

Bacteria	Group A (*n*)	Group B (*n*)	Group C (*n*)
*Staphilococcus pseudintermedius*	8	7	7
*Staphilococcus spp.*	5	2	3
*Enterococcus spp.*	2	1	–
*Streptococcus spp.*	–	2	–
*Proteus mirabilis*	2	–	1

## 4. Discussion

This study has shown FLE represents an applicable and well accepted option for the management of SBF in dogs. It significantly shortens the time to clinical resolution of SBF in comparison to systemic antibiotics, irrespective if administered once or twice weekly.

SBF, as part of bacterial pyoderma, is more common in dogs than other mammalian species and this fact has stimulated the research on this topic. Treating SBF usually requires a multimodal approach (19). Proper identification and treatment of the primary cause is mandatory since if only symptomatic medications are given, incomplete healing or recurrence is likely to occur (19).

Systemic antimicrobial drugs (AMD) are routinely used for the treatment of SBF (20). Selection of systemic AMDs is based on different criteria such as patient-specific factors (concurrent disease or drug administration, previous drug reactions, etc.), availability, safety, cost, and local incidence of resistant staphylococci (20). The continued reliance on commonly used empirical AMDs for the treatment of clinical symptoms attributable to SBF, especially in some geographic areas and including first-generation cephalosporins and beta-lactams, without a proper diagnostic workup including culture and sensitivity testing, is no more acceptable (21). Antibiotic resistance represents a global threat concerning human and animals (22). Veterinary dermatology is a high-consuming antibiotic branch and beta-lactam antibiotics have been frequently and empirically prescribed as first line therapy contributing to the increase over the last decades of MDR resistant infections (22–24).

In view of increasingly resistant bacterial strain identified in SBF, it is becoming critical to include topical therapy as a part of the symptomatic treatment of SBF. Topical antimicrobial treatment (such as shampoos, lotions, sprays, conditioners, ointments, baths, and other topical products) has been tested as sole treatment for superficial pyoderma, but many cases still necessitate or are currently treated with systemic antibiotics (20, 25). Topical therapies for SBF have been tested to produce significant potential advantages: (i) direct removal of causative microorganisms and debris of all their by-product from the cutaneous surface, (ii) minimal adverse effects and greatly reduced exposure to antimicrobial drugs (AMDs) of bystander organisms in other body district (reducing risk of inadvertent emergence of resistant strains), (iii) when deemed necessary, decrease of the duration of antimicrobial therapy length (26). Topical therapies, including use of antimicrobial devices (instead of drugs) and FLE, support as far as possible all the antibiotic stewardship programs (11) by optimizing the reliance on antibiotics only to selected infections and when the results of diagnostic testing are available.

In the present study the control group was managed only with oral antibiotic since authors believe this is a clear reflection of real life, for which the debate has not come to an end. The current literature indicates that the ordinary treatment duration for SBF resolution varies from 4 to 6 weeks (9). FLE has been showed to significantly accelerate the healing process of SBF when administered either once or twice weekly by greatly reducing the time needed to gain resolution with antibiotics. Consequently, even if proper assessment would have been appreciated, it is possible to speculate that FLE may consent a shorter duration of AMD therapy, when antimicrobial drugs cannot be avoided as already tested for deep and interdigital pyoderma, possibly improving patient outcomes, and decreasing the risk of AMD-related side effects (15, 16).

A very recent study has evaluated the caregiver burden, or strain from the challenges of providing care, for the owner of companion animal associated with skin disease (27). This charge is linked with loss of compliance and negative therapeutical plan outcomes (25), which happens quite frequently in dermatological diseases, and lower quality of life for both pet and owner (28–30). As a confirmation of this, two owners from the present study were incompliant with the proposed scheme and their dog withdrawn. Owners’ compliance is crucial to the resolution of SBF and prevention of recurrence. FLE regimen is applied in a veterinary-controlled environment thus allowing a constant evaluation of disease progression and treatment outcomes, while respecting owner’s assets. In addition, FLE supports owners to promote effective compliance by relieving them to the administration of home therapies (31–33).

The study, nonetheless, present some limitations including (i) the absence of a validated scoring system to assess the severity of SBF, (ii) the lack of quality of life (QoL) evaluation in dogs and their owners, and (iii) the small number of enrolled dogs. All these aspects deserve to be focused in further studies.

## 5. Conclusion

This study provides evidence that FLE may represent an effective therapeutical modality for superficial bacterial folliculitis in dogs, accelerating the time to clinical resolution and consequently reducing the duration of, or need for, systemic antibiotic treatment.

## Data availability statement

The raw data supporting the conclusions of this article will be made available by the authors, without undue reservation.

## Ethics statement

The animal study was reviewed and approved by University of Camerino Body for Protection of Animals. Written informed consent was obtained from the owners for the participation of their animals in this study.

## Author contributions

AM and AS: conceptualization and methodology. AM, AF, MC, and AS: investigation. AF and MC: data curation. AM, AF, and MC: writing—original draft preparation. AS: writing—review and editing. All authors contributed to the article and approved the submitted version.

## Conflict of interest

AM has received an honorarium from Vetoquinol, not related to the topic of this paper.

The remaining authors declare that the research was conducted in the absence of any commercial or financial relationships that could be construed as a potential conflict of interest.

## Publisher’s note

All claims expressed in this article are solely those of the authors and do not necessarily represent those of their affiliated organizations, or those of the publisher, the editors and the reviewers. Any product that may be evaluated in this article, or claim that may be made by its manufacturer, is not guaranteed or endorsed by the publisher.
